# Extremely potent immunotherapeutic activity of a STING agonist in the B16 melanoma model in vivo

**DOI:** 10.1186/2051-1426-1-S1-O15

**Published:** 2013-11-07

**Authors:** Leticia Corrales, Seng-Ryong Woo, Thomas F Gajewski

**Affiliations:** 1 Pathology, University of Chicago, Chicago, IL, USA; 2 Medicine, University of Chicago, Chicago, IL, USA

## 

We previously have reported that innate immune sensing of tumors in vivo involves type I IFN production by dendritic cells (DCs), which in turn acts on CD8α+ DCs to promote endogenous cross-priming of CD8+ T cells in vivo. Recently, we have identified the cytosolic DNA sensing pathway involving the molecule STING as the major upstream mechanism that leads to type I IFN production and DC activation in the setting of tumor implantation in vivo. Since most spontaneous T cell priming against tumors in vivo is suboptimal, ultimately ineffective, and fails to cause tumor rejection, we reasoned that stronger activation of the STING pathway could have therapeutic efficacy. To test this notion, we utilized DMXAA (5,6-dimethylxanthenone-4-acetic acid), a xanthenone derivative recently shown to directly bind to murine STING. In vitro, DMXAA led to STING aggregation as assessed by ImageStream cytometry, and induced phosphorylation of the downstream signaling proteins TBK1 and IRF3. Functionally, this led to the production of not only IFN-β, but also IL-6, TNF-α, and IL-12, as well as upregulated expression of MHCII, CD40 and CD86 by DCs. All of these functions were ablated when STING-/- DCs were treated. We therefore developed therapeutic regimens for in vivo studies using pre-established B16.SIY melanoma tumors. Surprisingly, a single intratumoral dose of DMXAA led to complete elimination of tumors in the majority of mice. The endogenous T cell response against SIY, as assessed by IFN-β ELISPOT and SIY/Kb pentamer FACS, was markedly increased in animals treated with DMXAA. This therapeutic effect was eliminated in STING-/- hosts. Mice with complete responses were completely protected against rechallenge with B16.SIY, suggesting immunologic memory. Slightly inferior but similar anti-tumor activity was observed with systemic (intraperitoneal) administration of DMXAA. Our data suggest that STING agonists can have profound therapeutic efficacy through the activation of host DCs and augmented priming of endogenous anti-tumor T cells in vivo. Inasmuch as DMXAA binds to murine but not human STING, the development of analogs that similarly engage human STING should receive high priority for ultimate clinical translation.

